# The Role of Metabotropic Glutamate Receptor 5 on the Stromal Cell-Derived Factor-1/CXCR4 System in Oral Cancer

**DOI:** 10.1371/journal.pone.0080773

**Published:** 2013-11-13

**Authors:** Nobuyuki Kuribayashi, Daisuke Uchida, Makoto Kinouchi, Natsumi Takamaru, Tetsuya Tamatani, Hirokazu Nagai, Youji Miyamoto

**Affiliations:** Department of Oral Surgery, Subdivision of Molecular Oral Medicine, Division of Integrated Sciences of Translational Research, Institute of Health Biosciences, The University of Tokushima Graduate School, Kuramoto, Tokushima, Japan; Winship Cancer Institute of Emory University, United States of America

## Abstract

We have demonstrated that blocking CXCR4 may be a potent anti-metastatic therapy for CXCR4-related oral cancer. However, as CXCR4 antagonists are currently in clinical use to induce the mobilization of hematopoietic stem cells, continuous administration as an inhibitor for the metastasis may lead to persistent leukocytosis. In this study, we investigated the novel therapeutic downstream target(s) of the SDF-1/CXCR4 system, using B88-SDF-1 cells, which have an autocrine SDF-1/CXCR4 system and exhibit distant metastatic potential *in vivo*. Microarray analysis revealed that 418 genes were upregulated in B88-SDF-1 cells. We identified a gene that is highly upregulated in B88-SDF-1 cells, metabotropic glutamate receptor 5 (mGluR5), which was downregulated following treatment with 1,1’ -[1,4-Phenylenebis(methylene)]bis-1,4,8,11-tetraazacyclotetradecane octahydrochloride (AMD3100), a CXCR4 antagonist. The upregulation of mGluR5 mRNA in the SDF-1/CXCR4 system was predominately regulated by the Ras-extracellular signal-regulated kinase (ERK)1/2 pathway. Additionally, the growth of B88-SDF-1 cells was not affected by the mGluR5 agonist (S)-3,5-DHPG (DHPG) or the mGluR5 antagonists 2-Methyl-6-(phenylethynyl)pyridine (MPEP) and 3-((2-Methyl-1,3-thiazol-4-yl)ethynyl)pyridine (MTEP). However, we observed that DHPG promoted B88-SDF-1 cell migration, whereas both MPEP and MTEP inhibited B88-SDF-1 cell migration. To assess drug toxicity, the antagonists were intraperitoneally injected into immunocompetent mice for 4 weeks. Mice injected with MPEP (5 mg/kg) and MTEP (5 mg/kg) did not exhibit any side effects, such as hematotoxicity, allergic reactions or weight loss. The administration of antagonists significantly inhibited the metastasis of B88-SDF-1 cells to the lungs of nude mice. These results suggest that blocking mGluR5 with antagonists such as MPEP and MTEP could prevent metastasis in CXCR4-related oral cancer without causing side effects.

## Introduction

We previously demonstrated that B88 cells, oral cancer cells that express the chemokine receptor CXCR4, specifically metastasize to cervical lymph nodes via a stromal cell-derived factor (SDF)-1 gradient produced by the lymphatic stroma [1-3]. The forced-expression of SDF-1 in B88 cells (named B88-SDF-1 cells) conferred enhanced cell motility and lung metastases following intravenous inoculation [4]. Recently, we also demonstrated that CXCR4 expression contributes to the metastatic potential of salivary gland cancers [5]. Furthermore, we have also demonstrated that blocking CXCR4 with 1,1’ -[1,4-Phenylenebis(methylene)]bis-1,4,8,11-tetraazacyclotetradecane octahydrochloride (AMD3100), a CXCR4 antagonist, may be a potent anti-metastatic therapy for CXCR4-related head and neck cancer [5,6]. 

SDF-1/CXCR4 system mainly functions as a chemotactic factor in cancer cells to reach metastatic sites. However, in order to establish the metastasis, several important processes, such as invasion, intravasation, extravasation and ectopic growth potential, are indispensable. Thus, it is critical to investigate the function of downstream target(s) responsible for the establishment of metastasis in SDF-1/CXCR4 system. Moreover, recent clinical trials have demonstrated that a single administration of AMD3100 is effective in mobilizing hematopoietic stem cells, despite the fact that AMD3100 is rapidly eliminated with an estimated distribution half-life of 0.3 hours and terminal half-life of 5.3 hours [7,8]. However, effective CXCR4-related anti-metastatic therapies may require the daily administration of AMD3100 to continuously prevent the migration of premetastatic cells to metastatic sites, which may cause chronic leukocytosis. If the downstream target gene(s) of SDF-1/CXCR4 system specifically expressed in cancer cells were identified, it is expected that anti-metastatic therapy might be performed more safely and effectively. However, the target gene(s) of SDF-1/CXCR4 system are not fully understood. Thus, in this study, we investigated the novel therapeutic downstream target(s) of the SDF-1/CXCR4 system in B88-SDF-1 cells, which have an autocrine SDF-1/CXCR4 system and exhibit distant metastatic potential in vivo, using cDNA microarrays [4]. 

## Materials and Methods

### Ethics statement

 The mice were handled in accordance with the recommendations in the Guide for the Care and Use of Laboratory Animals of the National Institutes of Health. The protocol was approved by the Committee on the Ethics of Animal Experiments of the University of Tokushima (Permit Number: 10084). All surgery was performed under sodium pentobarbital anaesthesia, and all efforts were made to minimize suffering. 

### cDNA microarray analysis

Total RNA for cDNA microarray analysis was extracted from serum-starved B88-mock cells and B88-SDF-1 cells. The Applied Biosystems Chemiluminescent RT-IVT Labeling Kit (Life Technologies, Carlsbad, CA, USA) was used to convert total RNA to digoxigenin (DIG)-labeled cRNA. One µg of total RNA was used to generate the double-strand cDNA. The cDNA was transcribed with DIG-labeled nucleotides (Roche Diagnostics, Basel, Switzerland), fragmented and hybridized to Human Genome Survey Array (Life Technologies) according to manufacturer’s instructions. After washing each array, the signal was developed with the use of a chemiluminescent detection kit (Life Technologies). Processed arrays were scanned with a 1700 chemiluminescent microarray analyzer (Life Technologies). These results were analyzed with the use of GeneSpring GX 12.5 (Agilent Technologies, Santa Clara, CA) and Ingenuity Pathway Analysis (IPA; Ingenuity® Systems, www.ingenuity.com, Redwood City, CA) software. Functional analysis of IPA identified biological functions or diseases that were most significant to data set. Fischer’s exact test was used to calculate a p-value determining the probability that each biological function or disease assigned to that data set was due to chance alone. The microarray raw data are deposited in Gene Expression Omnibus (GEO, http://www.ncbi.nlm.nih.gov/geo) according to minimum information about microarray experiment (MIAME) guidelines. The accession number is GSE50507. 

### Cells and cell culture

B88 cells were originally established from a patient with tongue cancer [1] and deemed free of mycoplasma and bacterial contaminants. Cells were maintained in Dulbecco’s Modified Eagle Medium (DMEM; Sigma, St. Louis, MO, USA) supplemented with 10% fetal calf serum (FCS), 100 µg/mL streptomycin, and 100 U/mL penicillin in a humidified atmosphere of 95% air and 5% CO2 at 37°C.

### Glutamate assay

A total of 5 x 10^6^ cells, were seeded on 100 mm dishes (Falcon; Becton Dickinson Labware, Franklin Lakes, NJ, USA). Medium was replaced with DMEM without L-glutamine and FCS after 24 h. After an additional 24 h culture, the conditioned medium was collected and 100 µl was analyzed with an Amplex® Red Glutamic Acid/Glutamate Oxidase Assay Kit (Life Technologies) according to the manufacturer’s instructions. Fluorescence was measured with a Varioskan Flash Multimode Reader (Thermo Fisher Scientific, Waltham, MA, USA) with 530 nm excitation and 590 nm fluorescence detection.

### Mice and in vivo study

C3H/HeN mice and BALB/c nude mice were purchased from CLEA Japan (Osaka, Japan) and were maintained under pathogen-free conditions. In the experimental chemotherapy, C3H/HeN mice were used as control immunocompetent mice [9], and were treated daily with either subcutaneous (s.c.) injections of AMD3100 (2.5 mg/kg; Sigma) [5,6] or intraperitoneal (i.p.) injections of 2-Methyl-6-(phenylethynyl)pyridine (5 mg/kg; MPEP; Tocris Bioscience, Bristol, UK) [10], 3-((2-Methyl-1,3-thiazol-4-yl)ethynyl)pyridine (5 mg/kg; MTEP; Calbiochem, San Diego, CA, USA) [10], or the same volume of saline. Mice treated with AMD3100 were sacrificed at day 1, 14 and 28, and mice treated with MPEP or MTEP were sacrificed at day 1, 7, 14, 21, and 28. All mice were sacrificed by exsanguination under sodium pentobarbital anesthesia and a complete blood count was performed using an ADVIA120 (Siemens Healthcare Diagnostics K.K., Tokyo, Japan) at Taiho Pharmaceutical Co., Ltd. (Tokyo, Japan). For the metastasis assay, 1 x 10^6^ B88-SDF-1 cells were inoculated intravenously (i.v.) prior to treatment with agents. Mice were sacrificed 30 days after cell inoculation, and lungs were extirpated and bisected. One half of the lung was fixed for histopathological analysis and stained with H-E and the other was lysed for quantitative analysis using Alu-PCR. The primers that were used for Alu-PCR were hAlu-UP: ACGCCTGTAATCCCAGCACTT, hAlu-DN: TCGCCCAGGCTGGAGTGCA [11]. Gene-specific products were measured continuously by an ABI PRISM 7000 Sequence Detection System for 35 cycles using THUNDERBIRD SYBR® qPCR Mix (TOYOBO, Osaka, Japan).

### RT-PCR

Cells cultured as monolayers were harvested at sub-confluence. After 24 h, RNA was isolated with TRIzol reagent (Life Technologies) according to the manufacturer’s instructions. RT-PCR for mGluR5 and glyceraldehyde 3-phosphate dehydrogenase (GAPDH) mRNA was performed under the following conditions: 94°C for 2 min; then 30 cycles of 94°C for 1 min, 60°C for 1 min and 72°C for 1 min; and a final extension at 72°C for 1 min. Primer sequences for human mGluR5 and GAPDH were as follows: mGluR5-UP: 5’-TGGCCACCCTGTTTGTTACT-3’, mGluR5-DN: 5’-GCACTGAGGCTGACCGAGAA-3’, GAPDH-UP: 5’-GAAATCCCATCACCATCTTCCAGG-3’, and GAPDH-DN: 5’-CATGTGGGCCATGAGGTCCACCAC-3’. For quantitative RT-PCR, we examined the expression of mGluR5 and GAPDH by a *Δ*ΔCT method. mGluR5 and GAPDH mRNA were simultaneously detected with Taqman^TM^ Gene Expression Assays (Life Technologies) according to the manufacturer’s instructions. Gene specific products were continuously measured by an ABI StepOnePlus Real-Time PCR System during 40 cycles of PCR.

### Flow cytometric analysis

Logarithmically growing cells were trypsinized and fixed in 4% paraformaldehyde (V/V) on ice for 10 min. The cells were washed and incubated with anti-mGluR5 mAb (dilution 1:100; R&D Systems, Minneapolis, MN, USA) for 30 min at room temperature. Cells were washed twice with D-PBS (-), then incubated with phycoerythrin (PE)-labeled goat anti-mouse IgG (Serotec, Sapporo, Japan) for 30 min at room temperature and analyzed with an EPICS flow cytometer (Coulter, San Jose, CA, USA).

### Immunofluorescent analysis

Cells were seeded on the Falcon CultureSlides (Falcon; Becton Dickinson Labware). Twenty hours later, cells were fixed with 4% paraformaldehyde for 10 min at room temperature. After washing the cells with PBS, non-specific binding was blocked with 1% BSA in PBS for 1 h at room temperature. Cells were then incubated with a primary rabbit monoclonal antibody against mGluR5 (GenWay Biotech, San Diego, CA, USA). Alexa Fluor 488-conjugated anti-rabbit IgG antibody (Life Technologies) was used for detection. Slides were counterstained with DAPI (Life Technologies) and mounted with ProLong Gold Antifade Reagent (Life Technologies). Fluorescence signals were observed with a confocal microscope (Nikon, Tokyo, Japan). For the detection of F-actin, B88-SDF-1 cells were incubated with either 100 µM mGluR5 agonist, (S)-3,5-DHPG (DHPG; TOCRIS) [12], 20 µM MPEP or 20 µM MTEP. After 24 h, cells were stained with Alexa Fluor 488-conjugated phalloidin (Life Technologies) and immunofluorescence was detected with an epifluorescence microscope (Nikon, Tokyo, Japan). 

### MTT assay

A total of 5 x 10^3^ cells were seeded in each well of a 96-well plate (Falcon; Becton Dickinson Labware) in DMEM supplemented with 10% FCS. After 24 h of culture, the cells were treated with either 100 µM DHPG, 20 µM MPEP or 20 µM MTEP for 48 h. The number of cells was quantified using the MTT assay [3-(4,5-dimethylthiazol-2-yl)-2,5-diphenyltetrazolium bromide, Sigma].

### Wound assay

After 24 h of culture, a linear wound was generated by scraping a confluent monolayers of cells with a pipet tip in the presence of either 100 µM DHPG or 20 µM MPEP or 20 µM MTEP. Unattached cells were washed off with agitation. Cells were imaged at the same grid location after 48 h. Each line was plated and wounded in triplicate. 

### In vitro cell migration assay

The *in vitro* migration of oral cancer cells was evaluated using a Transwell chamber (Corning, Corning, NY, USA). Cells in the membrane pores or cells attached to the lower surface of the membrane were counted in 10 fields of view at high magnification (x 400). In some experiments, 100 µM DHPG, 20 µM MPEP or 20 µM MTEP was added to the cells seeded on the upper chamber. 

### Statistical analysis

Statistical differences between the means values of the different treatment groups were evaluated with StatView 4.5 (Abacus Concepts, Berkeley, CA, USA) using a one-way ANOVA with the significance set at *p* < 0.05.

## Results

### Isolation of the target gene, metabotropic glutamate receptor 5, which is induced by the SDF-1/CXCR4 system

**Figure 1:**
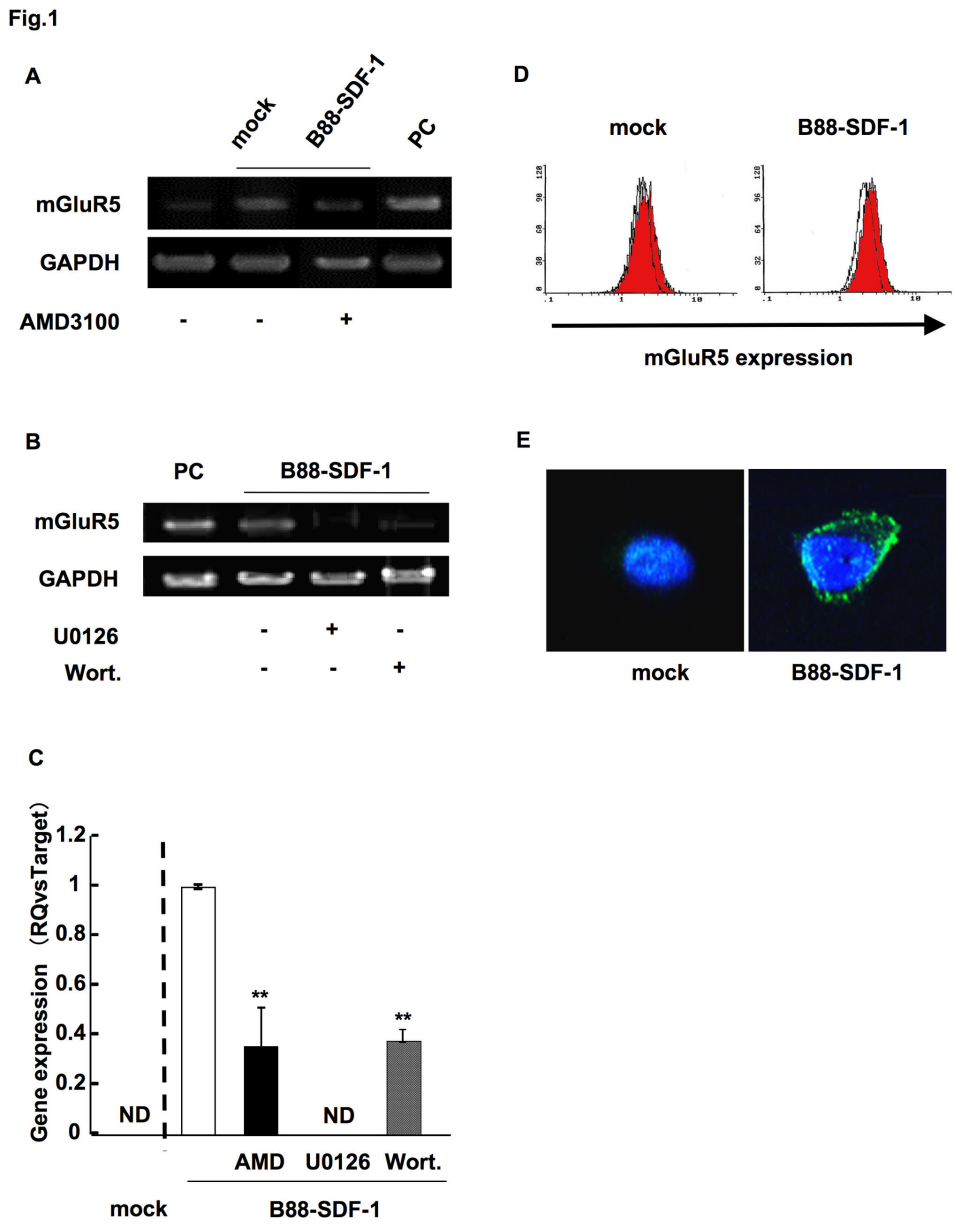
The upregulation of mGluR5 in B88-SDF-1 cells. (A) Expression of mGluR5 mRNA was confirmed in B88-mock and B88-SDF-1 cells in both the presence and absence of AMD3100 (1 µg/ml). Human placenta was used as a positive control (PC). (B) Cells were treated with U0126 (10 nM) or wortmannin (50 nM) for 48 h and mRNA expression of mGluR5 was analyzed by RT-PCR. (C) Expression of mGluR5 mRNA was confirmed by the real-time PCR. **; *p* < 0.01 when compared to untreated B88-SDF-1 cells by one-way ANOVA. ND; not detectable. (D) Protein expression of mGluR5 was evaluated in B88-mock and B88-SDF-1 cells using flow cytometry. Logarithmically growing cells were incubated with or without anti-mGluR5 mAb and stained with PE-labeled goat anti-mouse IgG. White and red zones indicate cells stained with the isotype control and the anti-mGluR5 mAb, respectively. (E) Protein expression of mGluR5 was detected by immunocytochemistry. The nucleus was stained with DAPI (blue).

We investigated novel therapeutic downstream target(s) of the SDF-1/CXCR4 system using the oral cancer cells, B88-SDF-1, which have an autocrine SDF-1/CXCR4 system and exhibit distant metastatic potentials *in vivo*. Microarray analysis revealed that 418 genes were upregulated in B88-SDF-1 cells compared to mock cells, and that the expression of metabotropic glutamate receptor 5 (mGluR5) increased 46-fold (5th from the top) in B88-SDF-1 cells, with the highest score corresponding to the mGluR5 pathway as shown by IPA (data not shown). Moreover, Park and colleagues demonstrated that strong mGluR5 expression is associated with patient survival and that mGluR5 antagonists inhibit the migration of oral cancer cells *in vitro* [13]. Thus, we analyzed mGluR5 as a possible candidate gene involved in the SDF-1/CXCR4 system. To confirm the specificity of the microarray analysis, the mRNA expression of mGluR5 was confirmed by RT-PCR. Similar to the microarray results, the mRNA expression of mGluR5 was upregulated in B88-SDF-1 cells, compared to mock cells (Figure 1A) and inhibited by treatment with AMD3100 (Figure 1A). We previously demonstrated that the SDF-1/CXCR4 system activates both the Ras-extracellular signal-regulated kinase (ERK)1/2 and the phosphatidylinositol 3 kinase (PI3K)-Akt pathways [1]. We therefore next examined the involvement of these pathways in the upregulation of mGluR5. The expression of mGluR5 was completely abrogated by treatment with U0126, a MEK inhibitor and partially inhibited with wortmannin, a PI3K inhibitor (Figure 1B). We also obtained the similar results in the quantitative RT-PCR (Figure 1C). Moreover, the upregulation of mGluR5 protein was also observed in flow cytometry and immunocytochemistry results (Figure 1D,E). 

### The expression of glutamate receptors in B88-SDF-1 cells

Glutamate receptors are divided into two categories; mGluRs and ionotropic GluRs (iGluRs), which are further characterized as either N-methyl-D-aspartate (NMDA), a-amino-3-hydroxy-5-methylisoxazole-4-propionic acid (AMPA) or kainate (KA) receptors [14,15]. We validated the expression of the glutamate receptors involved in the SDF-1/CXCR4 system using a cDNA microarray. Of the 8 types of mGluRs examined, only the expression of mGluR5 was markedly upregulated in B88-SDF-1 cells (Table 1). Furthermore, of the 14 types of iGluRs examined, the expression of GluR1, an AMPA receptor, increased 6-fold in B88-SDF-1 cells (Table 2). 

**Table 1:** Expression of mGluRs in cDNA microarray analysis.

Group	mGluRs	Fold induction*
I	mGluR1	1.27
	mGluR5	46.13
II	mGluR2	0.98
	mGluR3	0.67
	mGluR4	0.19
III	mGluR6	1.54
	mGluR7	1.06
	mGluR8	0.46

* Upregulation of B88-SDF-1 cells vs B88-mock cells

**Table 2:** Expression of iGluRs in cDNA microarray analysis.

Group	iGluRs	Fold induction *
AMPA	GluR1	6.05
	GluR2	1.42
	GluR3	ND
	GluR4	1.04
KA	GluR5	0.46
	GluR6	0.47
	GluR7	0.84
	KA1	0.41
	KA2	0.39
NMDA	NMDA1	2.34
	NMDA2A	2.21
	NMDA2B	0.71
	NMDA2C	0.12
	NMDA2D	1.23

* Upregulation of B88-SDF-1 cells vs B88-mock cells

### The production of glutamate in oral cancer cells

We next examined the production of glutamate, an mGluR5 ligand, in B88 and its transfectants, B88-mock and B88-SDF-1 cells. Glutamate production was detected in the conditioned media derived from these cells at a concentration of approximately 12 µM (Table 3). However, glutamate production was not dependent on either the SDF-1/CXCR4 system or the expression level of mGluR5. 

**Table 3:** Production of glutamate in oral cancer cells.

Cells	B88	B88-mock	B88-SDF-1
Glutamate release (μM)	12.23 ± 0.30	12.17 ± 0.17	12.21 ± 0.29

### The role of mGluR5 on cell growth

**Figure 2:**
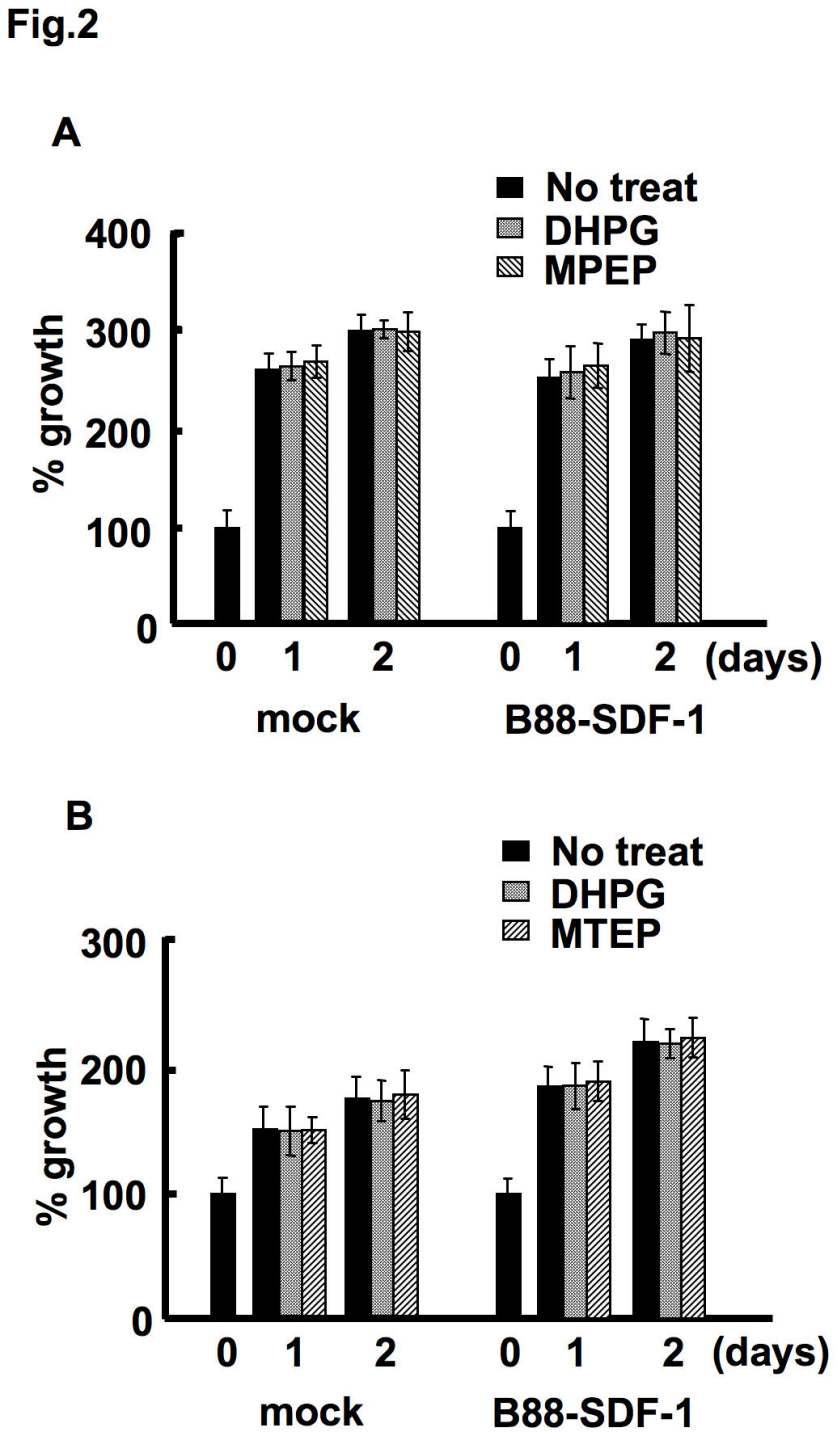
Cell growth was not affected by mGluR5. A confluent monolayer of cells was treated with either 100 µM DHPG, 20 µM MPEP (A) or 20 µM MTEP (B). The effect of mGluR5 on cell growth was evaluated using the MTT assay. There were no significant differences between the three groups by one-way ANOVA.

We examined the effect of mGluR5 on cell growth by using a specific mGluR5 agonist, DHPG, and two antagonists, MPEP and MTEP. The agonist and antagonists did not affect the growth of either the B88-mock or the B88-SDF-1 cells (Figure 2). 

### The role of mGluR5 on SDF-1/CXCR4-dependent cell migration

**Figure 3:**
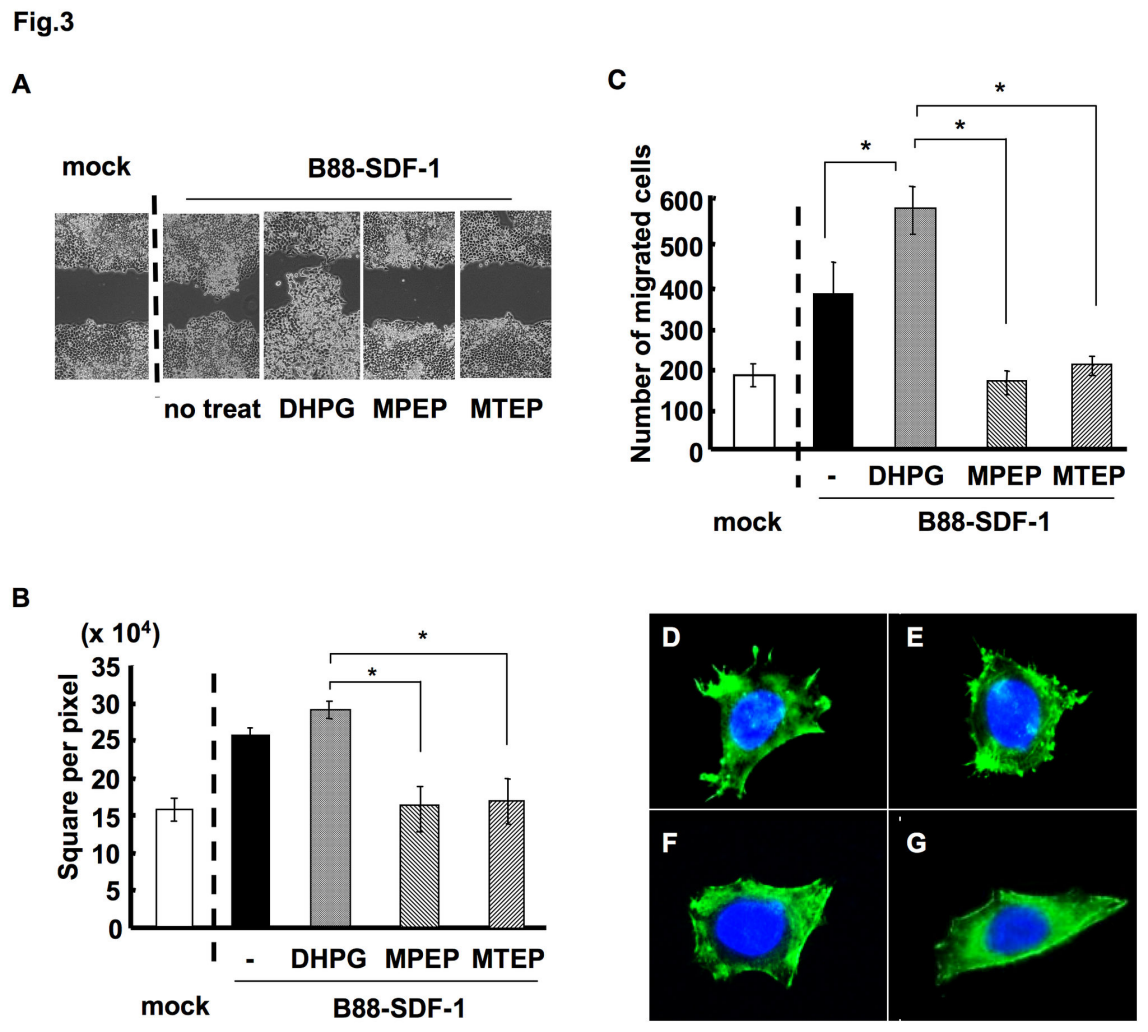
Involvement of mGluR5 on SDF-1/CXCR4-dependent cell migration. (A) B88-mock or B88-SDF-1 cells were cultured to confluence. A wound healing assay was performed in the presence of either 100 µM DHPG, 20 µM MPEP or 20 µM MTEP. (B) The quantitative data derived from (A). *; *p* < 0.05 when compared to DHPG-treated cells by one-way ANOVA. (C) The motility of B88-SDF-1 cells in the presence of either 100 µM DHPG, 20 µM MPEP or 20 µM MTEP was examined using a transwell migration assay. *; *p* < 0.05 when compared to untreated control or DHPG-treated cells by one-way ANOVA. (D-G) F-actin polymerization in the leading edge of B88-SDF-1 cells was examined by the immunocytochemistry following (D) no treatment, (E) DHPG treatment, (F) MPEP treatment and (G) MTEP treatment. Nucleus was stained with DAPI (blue).

We next investigated the effect of mGluR5 on the SDF-1/CXCR4-dependent migration of cells. Wound healing assays revealed that the enhanced motility of B88-SDF-1 cells was further accelerated with DHPG treatment, but was significantly impaired by MPEP and MTEP treatment (Figure 3A,B). Antagonists of mGluR5 also inhibited the migration of B88-SDF-1 cells, as shown by a migration chamber assay (Figure 3C). Furthermore, DHPG enhanced F-actin polymerization at the leading edge, whereas MPEP and MTEP inhibited F-actin polymerization (Figure 3D-G). We also observed the inhibition of matrigel invasion with MTEP treatment in B88-SDF-1 cells (data not shown).

### Effect of mGluR5 antagonists on immunocompetent mice

**Figure 4:**
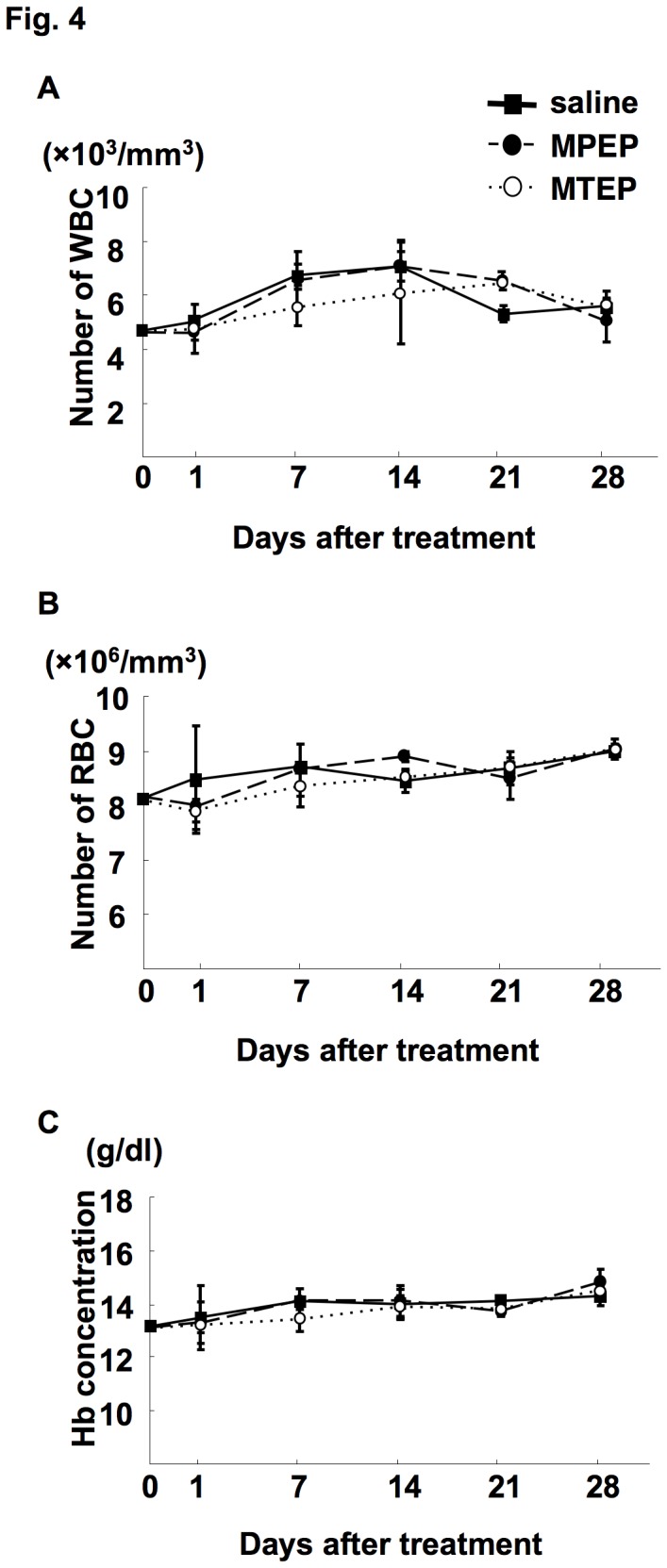
mGluR5 antagonists did not induce hematotoxicity. C3H/HeN mice were treated i.p. with MPEP (5 mg/kg), MTEP (5 mg/kg) or the same volume of saline daily. Mice were sacrificed on day 1, 7, 14, 21, and 28 by exsanguination and WBC (*A*), red blood cells (RBC; B) and hemoglobin (Hb; C) levels were measured. There were no significant differences between the three groups by one-way ANOVA.

Because mGluR5 may be a novel metastatic target in oral cancer, we evaluated the side effects of mGluR5 antagonists in C3/HeN mice. No weight loss or macroscopic organ abnormalities were detected in mice that were treated with the mGluR5 antagonists MPEP and MTEP (data not shown). Furthermore, these antagonists did not induce hematotoxicities such as anemia and leukocytosis (Figure 4A-C). We also evaluated the side effects of AMD3100 on C3H/HeN mice. No weight loss, macroscopic organ abnormalities or change in red blood cells count was observed in mice administered with AMD3100 (data not shown); however, significant and chronic leucocytosis was observed after daily s.c. administration of AMD3100 (data not shown). 

### The role of mGluR5 in the SDF-1/CXCR4-dependent cell metastasis

**Figure 5:**
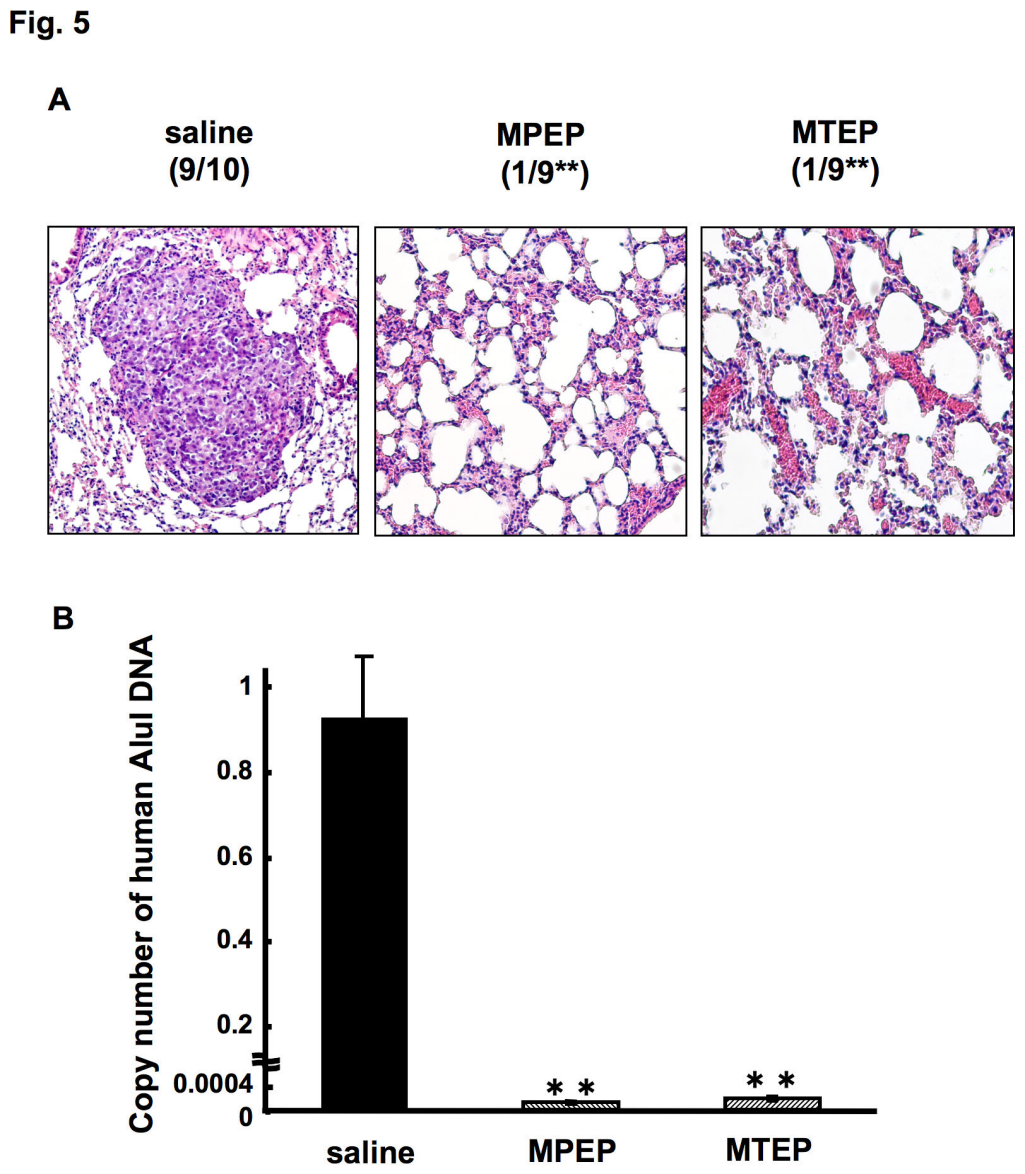
Inhibition of the SDF-1/CXCR4-dependent lung metastases by treatment with mGluR5 antagonists. Cells were inoculated into the blood vessel of nude mice, which were sacrificed on day 30. (A) Representative H&E staining of the lungs from control-treated (left), MPEP-treated (middle), MTEP-treated (right) nude mice. (B) Quantitative analysis by Alu-PCR was performed on the extirpated-lung tissues. **, *p* < 0.01 when compared to the saline control by one-way ANOVA.

Thus, we determined the effect of mGluR5 antagonists on lung metastases of B88-SDF-1 cells. Numerous metastatic nodules were detected in the lungs of mice that were inoculated with B88-SDF-1 cells (Figure 5A, left). However, a significant reduction in metastatic lung nodules was observed in mice treated with MPEP (Figure 5A, middle) and MTEP (Figure 5A, right), as shown by histopathological analysis during a 4 week observation period. We also confirmed the presence of metastatic cancer cells in extracted lung tissue using quantitative Alu-PCR. Consequently, the expression of human Alu DNA in mice treated with mGluR5 antagonists was significantly lower than in mice treated with saline (Figure 5B). 

## Discussion

Glutamate is a unique ligand for mGluR5, a metabotropic glutamate receptor belonging to the family of G-protein coupled receptors [16,17] that is ubiquitously found in the cerebral cortex, hippocampus, caudate nucleus and nucleus accumbens of the central nerve system (CNS) [16,17]. It has been suggested that mGluR5 is involved in CNS disorders that are induced by the hypersecretion of glutamate, such as epilepsy, neurogenic or inflammatory pain, psychosis, dyskinesia, headaches and drug addiction [16,17]. At the cellular level, mGluR5 regulates the growth and migration of glial cells [18], neural precursor stem cells [19], embryonic stem cells [20] and glioma cell [21]. Although the role of mGluR5 in cancer progression remains unclear, recent investigations suggest that mGluR5 functions in colon [22], breast [22] and prostate cancer cells [23]. Furthermore, Park and colleagues demonstrated that strong mGluR5 expression is associated with patient survival and that mGluR5 antagonists inhibit the migration of oral cancer cells *in vitro* [13]. These results suggest that mGluR5 functions as an oncogene in solid cancers, including oral cancer. On the other hands, recent investigations have demonstrated that glutamate is produced by mesenchymal cells, such as osteoblasts and osteoclasts, epithelial cells, such as pancreatic islet cells and keratinocytes, breast and prostate cancer cells [24-27]. Additionally, the production of glutamate in glioma cells has been reported to be associated with proliferation and invasion [21,28,29]. In addition, Bathe and colleagues demonstrated significantly increased concentrations of glutamate in the sera of patients with pancreatic cancer, as measured by metabolomics analysis [30]. In the present study, we demonstrate that B88 cells, and its transfected derivatives, produce glutamate in their conditioned media at a concentration of approximately 12 µM. These concentrations were similar to the findings described by Seidlitz and colleagues in the breast cancer cell line, MDA-MB-231 [29]. These results indicate that glutamate and its receptor are associated with cancer progression. However, no difference in glutamate production was observed between the B88-mock and B88-SDF-1 cells, although the expression of mGluR5, a receptor for glutamate, was upregulated in B88-SDF-1 cells. These results suggest that the expression of mGluR5, rather than glutamate production, is required for the glutamatergic system to be involved in SDF-1/CXCR4 signaling. 

Recently, synthetic mGluR5 antagonists have been developed as potential drugs for CNS disorders that are induced by the hypersecretion of glutamate [11,31,32]. It has been reported that administration of MPEP and MTEP has been used to effectively treat pain symptoms, Parkinson’s disease, cognition disorder, depression, anxiety, and schizophrenia [16,17]. Although we initially used MPEP as an mGluR5 antagonist in this study, significant non-specific actions of MPEP, including inhibition of AMPA or NMDA receptors, have been indicated [10]. Furthermore, we detected a 6-fold increase in the expression of GluR1, an AMPA receptor involved in the growth and invasion of glioma cells, in B88-SDF-1 cells [33,34]. To exclude the non-specific effect of MPEP on mGluR5, we re-evaluated the role of mGluR5 on the SDF-1/CXCR4 system using the recently developed mGluR5 antagonist MTEP, which is more highly selective for mGluR5 and has fewer off-target effects than MPEP [10]. However, both MPEP and MTEP had similar effects on the migration and metastasis of B88-SDF-1 cells, indicating a specific effect of mGluR5 on the SDF-1/CXCR4 system. 

We detected additive effects of mGluR5 agonists DHPG in migration assay in spite of containing a large amount of glutamate in the media. Although we did not observe the direct activation of mGluR5, it is considered that DHPG probably activate mGluR5 in B88-SDF-1 cells because DHPG did not enhance the migration in mock cells, which do not express mGluR5 (data not shown). Cleva and Olive demonstrated that mGluR5 are physically coupled to NMDA receptors by various scaffolding proteins, and are biochemically coupled to NMDA receptor function via PKC [35]. Furthermore, this mGluR5-NMDA interaction has been observed in numerous brain preparations, whereby activation of mGluR5 receptors with DHPG potentiates NMDA receptor-mediated responses to exogenously applied glutamate or NMDA [35]. Because B88-SDF-1 cells express NMDA receptors in our microarray analysis (Table 1), this additive effect of DHPG might be due to the concomitant activation of NMDA receptor pathway. 

In the present study, we demonstrated the involvement of the Ras-ERK1/2 pathway on the upregulation of mGluR5 by the SDF-1/CXCR4 system. Although an association between mGluR5 and the SDF-1/CXCR4 system has not been reported in cancer cells, Luo and colleagues have demonstrated the induction of mGluR5 on E14.5 neural precursor cells by stimulating with SDF-1. They also suggested that mGluR5 expression by the SDF-1/CXCR4 system is induced by the transcription factor, Ets, which is activated by the ERK1/2 signaling pathway [36]. Because the *mGluR5* gene has Ets binding sites in their promoters [37], the CXCR4/ERK1/2/Ets pathway might be involved in the induction of mGluR5 that was observed in our experiment. 

We did the same experiment using an oral SCC cell line, HNt, in which the expression of CXCR4 is 7.5-fold lower than that in B88 cells [1]. HNt-SDF-1 cells did exhibit slight, but not significant, phenotypic changes in vitro and in vivo [4], and the mGluR5 induction was detected only in a marginal level, probably due to the reduced expression of CXCR4, compared with that of B88 cells. Thus, CXCR4 expression level and strong downstream signaling might be also critical for the activation of mGluR5 pathway. 

We discovered that mGluR5 antagonists significantly inhibited SDF-1/CXCR4-dependent migration and metastasis. Although the SDF-1/CXCR4 system mainly functions as a chemotactic factor in cancer cells, it is also involved in the several metastatic processes, such as neovascularization, cell adhesion, invasion, outgrowth and epithelial to mesenchymal transition [38-43]. In the present study, mGluR5 regulated the cell migration associated with the SDF-1/CXCR4 system; however, it is unlikely that mGluR5 antagonists suppress SDF-1/CXCR4-dependent metastasis only via inhibited cell migration. Although mGluR5 has been shown to enhance the adhesion and invasion of oral cancer cells [13] and the outgrowth of neural cells [44], little information is available regarding metastasis-related functions. mGluRs activate both the MAPK and Akt pathways, which are two hallmark signaling pathways that promote cancer growth and metastasis [45,46]. Thus, targeting mGluR5 may suppress these critical metastatic pathways and inhibit cancer metastasis. 

In our previous study, we detected CXCR4 expression in approximately 60% of primary oral cancers and concluded that CXCR4-positive cases had a significantly worse prognosis than CXCR4-negative cases [3]. Although we did not examine the expression of mGluR5 in oral cancer tissues, Park and colleagues demonstrated that 70% of oral cancers express mGluR5 and that overexpression of mGluR5 decreases the survival rate of patients with oral cancer [13]. Taken together, the association between CXCR4 and mGluR5 is strongly suggested to promote the progression of oral cancer. To our knowledge, the mGluR5 antagonists MPEP and MTEP are not yet clinically available, even though no significant side effects were reported in the animal studies [10]. In the present study, the administration of mGluR5 antagonists did not cause weight loss or hematotoxicity in immunocompetent mice. Furthermore, because mGluR5 is involved in various diseases that are induced by the hypersecretion of glutamate, novel mGluR5 antagonists are currently being developed. If further molecular mechanism of mGluR5 against cancer metastasis could be clarified, blocking mGluR5 with antagonists such as MPEP and MTEP could prevent metastasis in CXCR4-related oral cancer without causing side effects.
